# The Platelet/Megakaryocyte Axis is Necessary for Allergic Sensitisation and Inflammatory Responses to House Dust Mite in the Lung

**DOI:** 10.1007/s00408-026-00896-w

**Published:** 2026-05-19

**Authors:** Anna Chalidou, Katie-Marie Case, Carl Hobbs, Clive P. Page, Simon C. Pitchford

**Affiliations:** 1 Pulmonary Pharmacology Unit, Institute of Pharmaceutical Science, King’s College London, Room 5.43 Franklin Wilkins Building, Waterloo Campus, London, SE9 1NH UK; 2 Wolfson SPaRC (Sensory, Pain and Regeneration Centre), Institute of Psychiatry, Psychology & Neuroscience, King’s College London, Guy’s Campus, London, SE1 1UL UK

**Keywords:** Platelets, Megakaryocytes, HDM, Sensitisation, IgE, IL-4, IL-13, MHC Class II, Antigen-presenting cells

## Abstract

**Background:**

Evidence demonstrates that megakaryocytes (MKs) possess antigen processing and presentation properties. We investigated whether sensitisation to house dust mite (HDM) extract and the establishment of allergic pulmonary inflammation was dependent on the presence of platelets, and whether the allergic phenotype had an effect on antigen-presenting marker expression on MKs and platelets.

**Methods:**

Balb/c mice were administered anti-GPIbα antibody or an isotype control during days 0, 2, and 4 to temporarily deplete circulating platelets during initial sensitization to allergen (HDM extract) via an extended intranasal administration on days 0–4, 7–11, and 13. Bronchoalveolar lavage was undertaken, along with blood samples, and lung histology to quantify leukocyte recruitment, Th2 cytokine analysis, and lung platelet deposition. Lung MKs, bone marrow MKs, and circulating platelets were analysed from both allergen-sensitized and non-sensitized mice for the expression of MHC class I, MHC class II, FcεRIα, and CD40.

**Results:**

Temporary platelet depletion limited to the initial phase of allergen sensitization inhibited the development of the allergic pulmonary phenotype to HDM as demonstrated by reduced eosinophil recruitment, IgE titre, IL-4 and IL-13 expression. Lung-resident MKs demonstrated higher expression of MHC class II and FcεRIα when compared to bone marrow (BM) MKs on establishment of an allergic phenotype, suggesting the administration of HDM specifically affected the immune-phenotype of the lung niche of MKs rather than the bone marrow niche, or systemic effects on circulating platelets. Platelets within the lung were observed to have increased co-localisation frequency with CD11c-positive antigen-presenting cells (APCs), but not CD4-positive T cells, suggesting that platelet activation may occur during the initial steps of allergen sensitisation.

**Conclusion:**

The platelet/MK axis is a requirement for sensitisation to HDM. An allergic phenotype selectively influences the immune signature of MKs in the lung.

**Supplementary Information:**

The online version contains supplementary material available at 10.1007/s00408-026-00896-w.

## Introduction

The activation of platelets in the establishment of sensitisation to allergens and the subsequent inflammatory response to allergen exposure in vivo has been demonstrated to be required for pulmonary eosinophil and lymphocyte recruitment, airway hyper-responsiveness, airway remodelling, induction of T-reg generation, and production of IgE and IL-4 [1–8]. The exposure of allergic subjects to *Dermatophaoides pteronyssinus* (house dust mite; HDM) induced changes in lung function associated with platelet activation [9, 10]. Furthermore, platelets interact with allergens in an IgE-dependent manner, with platelet recruitment to the lung being reliant on an IgE- FcεRIα interaction [11]. Increased pulmonary pools of both platelets and their progenitor cells, megakaryocytes (MKs) have been reported at autopsy in patients dying from *status asthmaticus* [12–14], and lung-resident MKs have been reported to produce platelets in vivo [15]. Single-cell transcriptomics have revealed cluster formation within the MK population according to their functionality including a MK subpopulation defined as ‘inflammatory response-associated MKs’ [16]. On comparison of transcriptomic profiles of MKs resident in the lung versus the bone marrow (BM), the former were found to be selectively enriched for genes and pathways relevant to immunoregulatory functions [15]. Interestingly, the transcriptomic landscape of lung-resident MKs was found to be strikingly similar to that of lung dendritic cells (DCs) [17]. This observation was additionally supported by flow cytometric studies demonstrating lung MK-restricted expression of MHC class II and higher levels of expression of co-stimulatory molecules (CD86, CD80 and CD40) involved in the process of antigen presentation [17]. Lung resident MKs were demonstrated to process antigen to CD4-positive (CD4+) T cells in a MHC class II dependent manner [17]. Conversely, antigen processing in murine BM resident MKs was demonstrated to be dependent on a MHC class I process, where MKs were not only able to load and present the antigen on their surface, but also pass the antigen- MHC class I complexes to proplatelets [18]. Platelets expressing MHC class I proteins can also internalise and present antigens, with the protein expression levels correlating to sepsis-associated mortality in vivo [19]. These studies reveal compelling mechanisms by which MKs and/or platelets can process antigen in vitro. However, since MKs from both BM and lung niches produce platelets, it is possible that allergen sensitisation is driven primarily by resident MKs or by their platelet progeny, which can be produced in the lung vasculature and accumulate in the tissue. Since platelets lack nuclei and possess no transcriptional capabilities, they inherit their transcriptomes and proteomes from the respective progenitor MKs, suggesting generation of platelet subpopulation pools capable of differential interaction with components of the adaptive immune system as part of the antigen processing and presentation events.

Here we investigated whether the sensitization and development of the allergic pulmonary phenotype towards HDM extract exposure was dependent on the presence of platelets that may be progeny from either lung, or BM residing MKs that have the subsequent ability to migrate into the lung. Furthermore, we investigated the expression of proteins involved in allergen recognition and processing on MKs taken from different organ niches and circulating platelets on the establishment of a Th2-type immune response.

## Materials and Methods

Female wild-type BALB/c mice (6–8 weeks of age) were purchased from Charles River, UK. Female mice were previously reported to respond well to allergen [14, 20]. It should be noted however that gender differences may exist with regard to platelet activation, with reported activity higher in (human) females, although other studies do not reveal a difference [21–23]. The animals were randomly allocated into groups upon arrival and group-housed with 4–6 mice per cage in a temperature and humidity-controlled environment with a 12-hour light-dark cycle. Food and water access were provided *ad libitum*. All experiments were conducted in compliance with the Animals (Scientific Procedures) Act (ASPA, 1986), the Animal Welfare and Ethics Committee at King’s College London, and Animal Research: Reporting In Vivo Experiments (ARRIVE) guidelines 2.0 [24]. Animals were culled by anaesthetic overdose (0.2 ml of 25% urethane, intraperitoneally) on days 5 and 14, 24 h after the last intranasal (i.n.) administration of HDM extract (below).

### Sensitisation Protocol to HDM Extract Containing *Der P I* Allergen

House dust mite (*D. Pteronnyssinus;* HDM) extract was purchased from Greer Laboratories (XPB80D3A2.5) and formulated at 1 mg/ml of total protein (HDM extract containing *Der P I* allergen at 54 µg of *Der P I* per 1 mg of total protein) in sterile filtered 1 x phosphate buffered saline (PBS) and aliquoted to be stored at −20 °C. Allergen aliquots were thawed on the day of use, 20 min prior to treatment initiation. Vehicle (1x PBS) was used as a negative control for allergen administration. Animals were administered PBS or HDM (i.n. 25 µl) on days 0–4, 7–11 and 13 under light isoflurane anaesthesia (4.0–4.5% at 2 L/min O_2_), following previously published protocols [14, 20].

### Treatment Regimen–Platelet Depletion Protocol

A purified rat monoclonal anti-mouse anti-platelet antibody (anti GP1bα -R300 Emfret) was used to deplete platelets. The antibody preparation contains a mixture of purified rat monoclonal antibodies directed against mouse GPIbα. Targeting of this receptor with divalent IgGs results in profound and irreversible Fc-independent platelet depletion in mice that is platelet specific [25, 26]. Mouse IgG2a isotype control antibody (BioXCell, BE0085) was administered to control groups of mice. Both antibody preparations were formulated fresh at 0.2 mg/ml in sterile filtered 1xPBS prior to each dosing occasion. Treatments were administered intravenously (i.v.) at a dose of 1 mg/kg and dose volume of 5 ml/kg on days 0, 2 and 4, one hour prior to the i.n. HDM extract administration to induce temporary thrombocytopenia restricted to the sensitisation phase.

### Analysis of Tissues, Cells and Biochemical Analysis

Blood samples were collected to examine changes in platelet and leukocyte numbers, and total plasma IgE levels over the time course of allergen exposure. Bronchoalveolar lavage fluid (BALF) was harvested to enumerate leukocyte numbers and to quantify levels of IL-4, IL-13 and IL-33 on days 5 and 14. Blood samples and single-cell suspensions generated from the right lung and BM were used for flow cytometry to assess surface protein expression (MHC class I, MHC class II, FcεRIα, and CD40) on MKs. Left lungs were used for fluorescent immunohistochemistry to quantify CD41, CD4 and CD11c positive events, and their co-localisation per mm^2^ of lung tissue. Lung-draining lymph nodes were used to quantify the frequency of CD42b-positive events in the lung. The experimenter was blinded to the identity of samples during enumeration of platelet and leukocyte numbers in blood and BALF, and quantitative immunohistochemistry. Detailed description of the methodology of the flow cytometric protocols, histopathological examination of tissue, collection and enumeration of BALF and biochemical assays can be found in the online supplementary materials and follow previously published protocols [27–29].

### Statistical Analysis

Data are expressed as mean +/− SEM are biological replicates (i.e. each sample is obtained from an individual mouse). Flow cytometry data was analysed with a commercially-available software (FlowJO; v10.8.1). Histopathological analysis was performed in ImageJ (1.54f). When comparing the number of platelets and leukocytes in the blood or BALF, ELISA results and organ weights, a 2-Way ANOVA (analysis of variance) with Fisher’s LSD (least significant difference) post hoc test was used (*p* < 0.05). When comparing surface protein expression on the surface of blood platelets, a Mann-Whitney test was used (*p* < 0.05). When comparing surface protein expression on the surface of lung and BM MKs, a 2-Way ANOVA with Fisher’s LSD post hoc test was used (*p* < 0.05). When comparing the number of events positive for a marker utilising immunohistochemistry (IHC) or immunofluorescence (IF) staining techniques, a Mann-Whitney test was used (*p* < 0.05). All statistical analyses were performed in GraphPad Prism (v10).

## Results

### Platelet Depletion Prevented the Development of an Allergic Pulmonary Phenotype to *Der P I* Allergen In Vivo

**Fig. 1:**
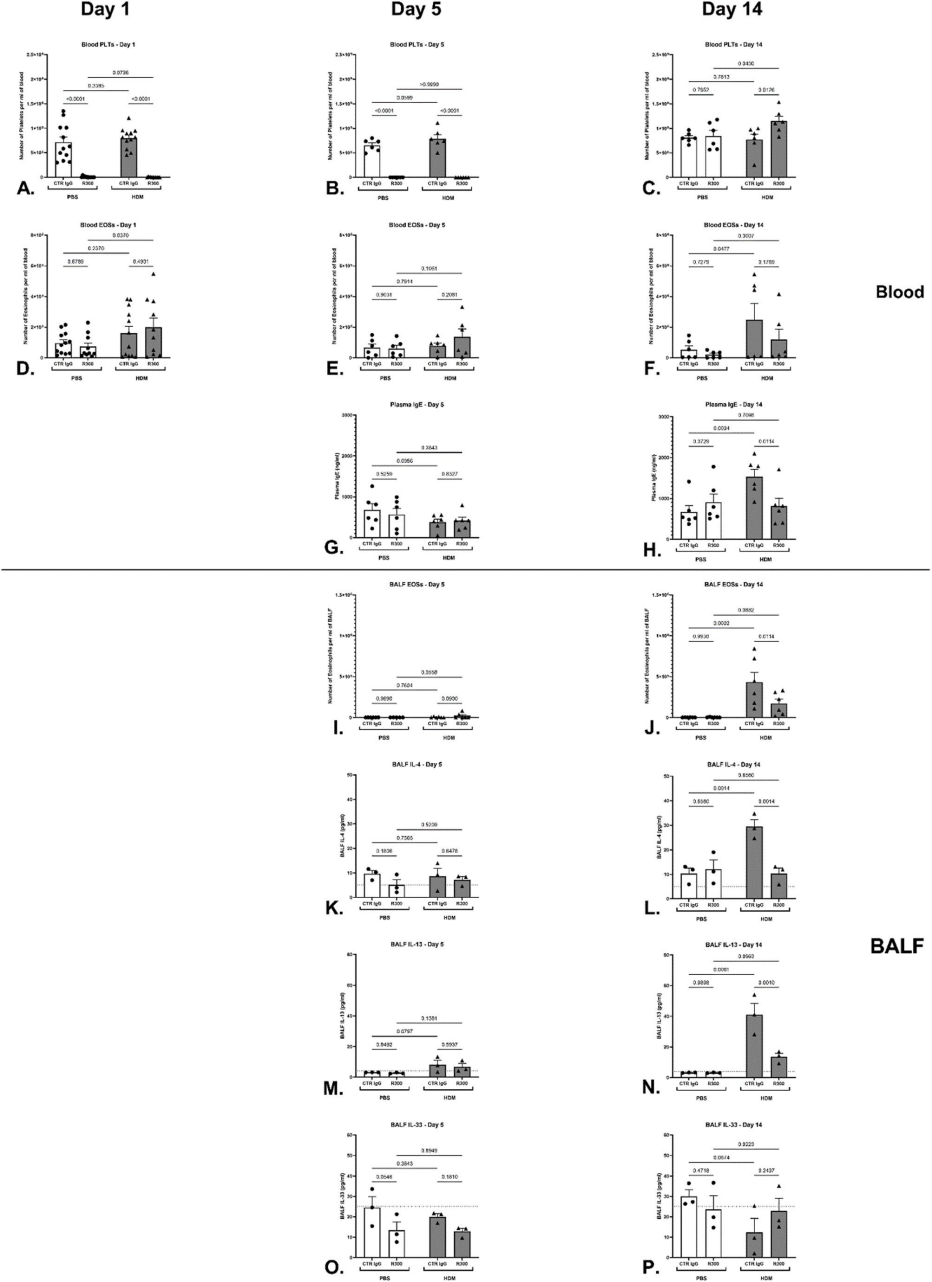
Time course of systemic and local immune changes following platelet depletion and allergen sensitisation. **(A-C)** Circulating platelet counts of Days 1, 5 and 14 (*n* = 12, 6, and 6 per group, respectively). **(D-F)** Circulating eosinophil counts of Days 1, 5 and 14 (*n* = 12, 6, and 6 per group, respectively). **(G-H)** Total plasma IgE levels on Days 5 and 14 (*n* = 6 per group). **(I-J)** Eosinophil numbers in BALF on Days 5 and 14 (*n* = 5–6 and 9 per group, respectively). Concentrations of IL-4, IL-13 and IL-33 in BALF on Day 5 **(K**,** M**,** O)** and Day 14 **(L**,** N**,** P)** (*n* = 3 per group). Samples on Day 1 and 5 were collected 24 h after the last R300 dose administration (i.v.). Samples on Day 14 were collected 24 h after the last HDM dose administration (i.n.). Data presented as mean +/− SEM (*p* < 0.05, 2-Way ANOVA with Uncorrected Fisher’s LSD). Data is from two independent experiments for blood, BALF and serum IgE data, and one experiment for cytokine measurements. Each data point represents an individual mouse

Although the effect of experimentally induced thrombocytopenia on the development of the allergic phenotype in vivo has been studied before, no studies have investigated the importance of platelets at the time of sensitisation to a clinically relevant allergen. To confirm the successful induction of experimentally induced temporary thrombocytopenia via administration of the anti-GP1bα antibody, during the period of initial exposure to *Der P I* allergen from HDM extract, tail vein blood samples were used to quantify platelet numbers on days 1 and 5, 24 h after the last administration of the anti-GP1bα antibody, and on day 14, 24 h after the last administration of HDM extract. Platelet depletion by over 95% was successfully induced throughout the first phase of the experiment and successfully recovered to normal circulating levels on cessation of administration of the anti-GP1bα antibody as assessed on day 14 (Fig. 1A–C).

Assessment of establishment of the allergic pulmonary phenotype demonstrated that no representative biomarkers – blood and BALF eosinophilia, total plasma IgE, BALF IL-4, IL-13 and IL-33 - were elevated on day 5 in the HDM extract -treated animal groups of animals rendered temporarily thrombocytopenic during the sensitisation phase (Fig. 1E, G, I, K, M, O respectively). Additionally, only animals in the combination treatment group of control IgG and HDM extract exposure developed an allergic pulmonary phenotype as reflected by the increase in blood and BALF eosinophil numbers (Fig. 1F, J), total plasma IgE (Fig. 1H) and BALF IL-4 and IL-13 (Fig. 1L, N). Collectively, these data demonstrate that the allergic pulmonary inflammation phenotype was only established by day 14, therefore confirming that day 5 as an earlier time-point is representative of the sensitisation phase to the allergen when temporary platelet depletion was induced.

Platelet depletion during the sensitisation phase to HDM extract prevented the development of the subsequent allergic pulmonary phenotype as demonstrated by significantly reduced levels of blood and BALF eosinophils (Fig. 1F, J), total plasma IgE (Fig. 1H), and the lack of upregulation of BALF IL-4 and IL-13 (Fig. 1L, N) in comparison to the positive control group.

(Ctr IgG/HDM extract), and with similarity to the respective non-sensitized control groups. Importantly, the administration of the anti-GP1bα antibody had no effect on any circulating blood leukocyte subsets, further demonstrating the selectivity of this methodology (Supplementary Fig. 1).

**Fig. 2:**
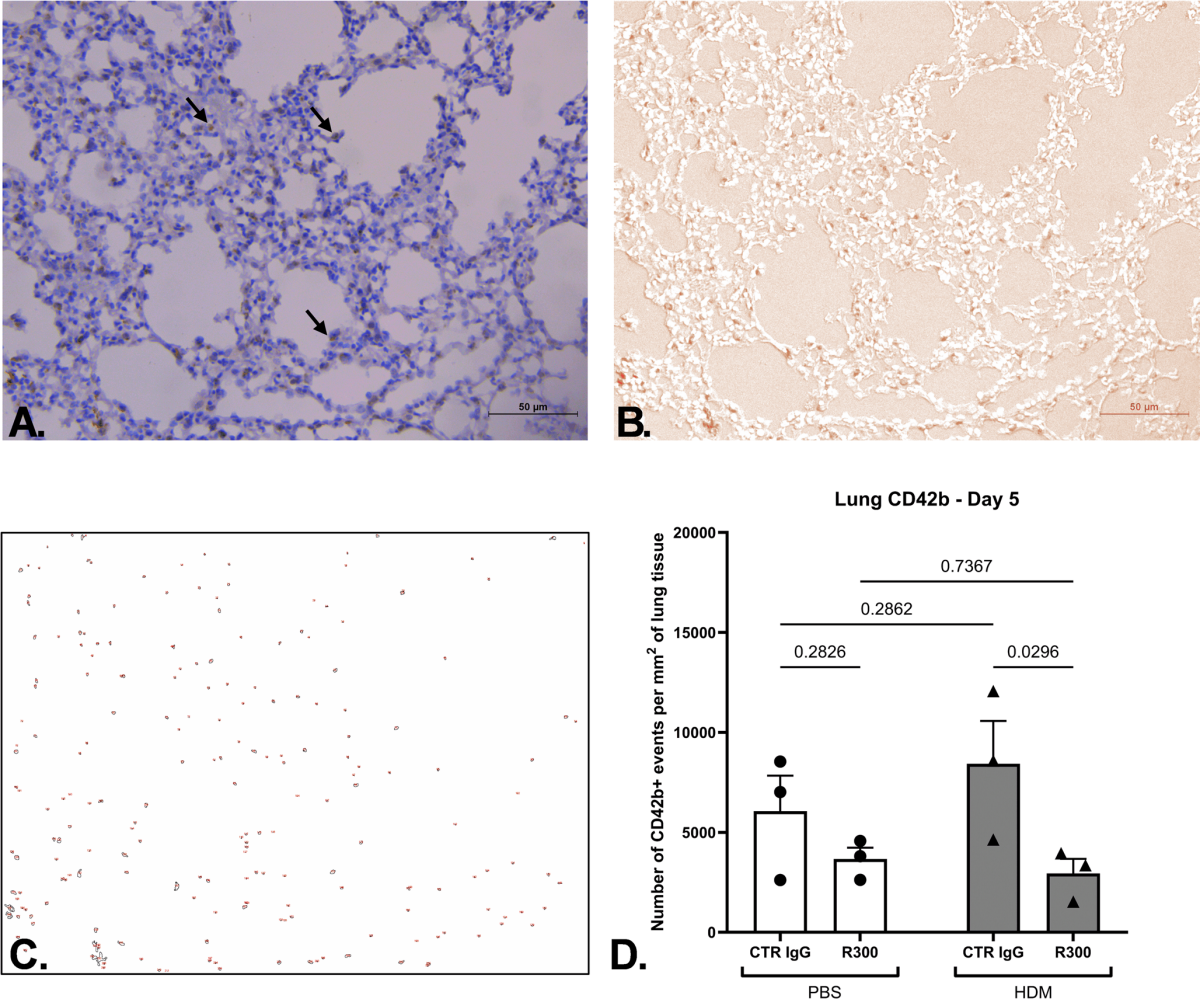
Presence of platelets in lung tissue. Platelets were detected utilising the DAB staining technique for the CD42b marker expression Day 5 (*n* = 3 per group) Lungs on Day 5 were excised 24 h after the last intravenous dose administration. Six fields of view at 20x magnification were selected in a random manner, and a mean value for each animal tissue was calculated. Visual platelet identification **(A)**, separated brown DAB signal through the utilisation of the colour deconvolution plugin (**B**), application of colour threshold discriminator to separate signal from noise (**C**), quantification (**D**). Each datapoint corresponds to the individual animal mean for the respective readout. Data presented as group mean (*p* < 0.05, 2-Way ANOVA with Uncorrected Fisher’s LSD). Data obtained from one experiment. Each data point represents an individual mouse

Enumeration of platelets present in lung tissue was assessed by immunostaining lung slices with an anti-CD42b antibody (Fig. 2A–C). Positive events were differentiated from MKs via controlling for nuclear counterstaining. When assessed on day 5, 24 h after the last intranasal administration of HDM extract, animals that had been treated with anti-GP1bα antibody (R300/HDM combination group) demonstrated significantly lower platelet numbers in the lung tissue when compared to the respective positive control group (Ctr IgG/HDM extract) (Fig. 2D). These data demonstrate that the systemic administration of the platelet-depleting antibody not only had an effect on the number of circulating blood platelets, but also affected platelets located within the lung.

### HDM Extract -Induced Allergic Pulmonary Inflammation Differentially Changes the Immunophenotype of MKs Depending on the Organ Niche In Vivo

Previous studies report immunophenotypic differences between lung and BM-resident MKs, with further evidence demonstrating the ability of lung-resident MKs to produce platelets. Here we aimed to examine whether the sensitisation to HDM extract influenced the surface marker expression of proteins relevant to antigen processing and presentation as well as determine whether these occurred differentially depending on the organ niche.

**Fig. 3:**
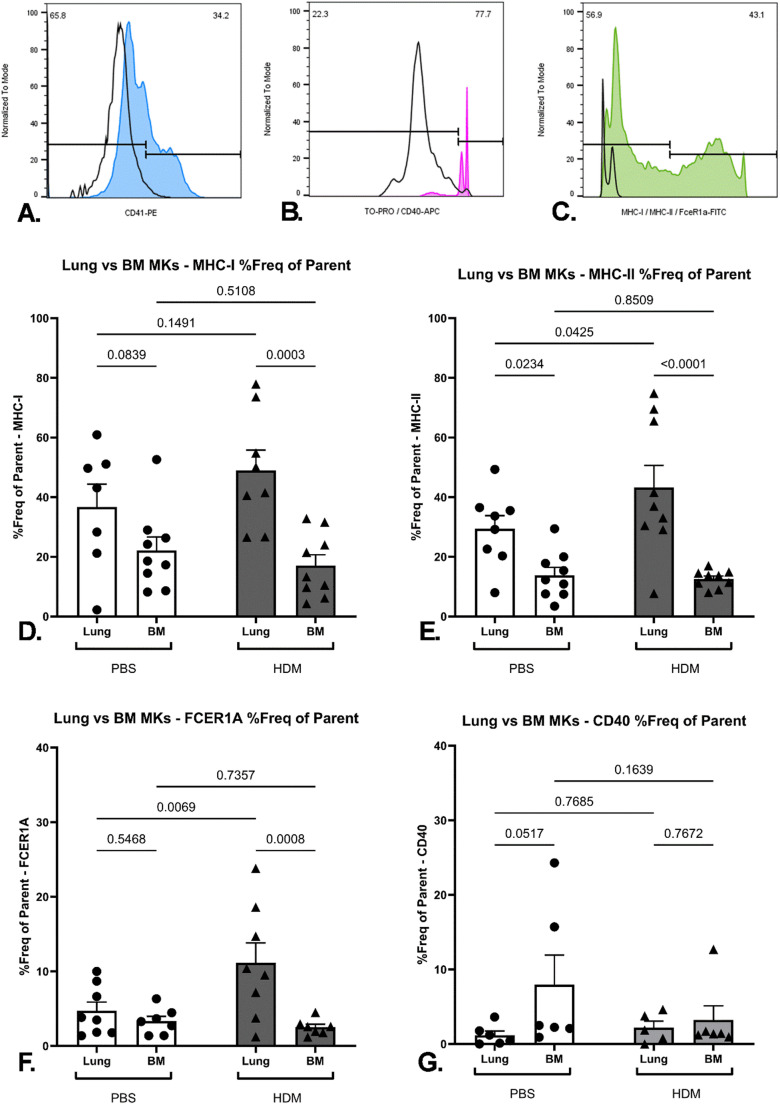
Flow cytometric expression of surface antigens on megakaryocytes. Cells were identified using forward scatter-area (FSC-A) versus side scatter area (SSC-A) axes is subsequently examined for **(A)** PE-CD41 positivity (blue histogram) against a triple isotype control staining condition of the same sample (black solid line) and next for **(B)** APC-TOPRO-3 positivity (magenta histogram) against a double-isotype control staining condition (black solid line) followed by a **(C)** FITC- MHC class I, MHC class II or FcƐRIα, or APC-CD40 antibody (green histogram) against a double isotype control staining condition of the same sample (black solid line). Percentage of cells positive for: MHC class I (*n* = 7–9 per group) (**D**), MHC class II (*n* = 8–9 per group) (**E**), FceRIα (*n* = 7–8 per group) (**F**), and CD40 (*n* = 5–6 per group) (**G**) on the surface of lung and bone marrow-resident MKs. Tissue samples were collected and processed to generate single-cell suspensions on Day 14, 24 h after the last PBS or HDM extract dose administration. Data expressed as mean +/− SEM (*p* < 0.05, 2-Way ANOVA with Uncorrected Fisher’s LSD). Data is from two independent experiments, except for CD40 expression, which was obtained from one experiment. Each data point represents an individual mouseCollectively, these data demonstrate that the organ niche is a central determinant of surface protein expression of MKs pertinent to allergen presentation and processing. This suggests that MKs might potentially possess diverse roles in terms of inflammatory response and haematopoiesis. Lung-resident MKs (or their platelet progeny) are more likely to play a direct role in the propagation and establishment of the allergic pulmonary phenotype to HDM extract in vivo, as they possess the protein repertoire required for antigen presentation and direct interaction with IgE antibodies

MHC class I, MHC class II, FcεRIα and CD40 expression were examined on MKs via flow cytometric methods. MKs were identified in the single-cell suspensions prepared from lung and BM tissue through identifying the CD41 and TOPRO-3 double-positive events, to confirm lineage and ploidy (Fig. 3A–C). This strategy allowed us to confirm that the ploidy content of CD41-poitive cells was significantly higher, and in the ≥4n ploidy region that is indicative of MKs with few cells in the 2n ploidy region [17, 30]. This is compared to CD41-negative cells where a mixture of 2n and 4n events is evident (Supplementary Fig. 2A–C). Analysis of the TOPRO staining on CD41-negative versus CD41-positive cellular events revealed a significant increase in mean fluorescent intensity of signal as befits this increased cellular ploidy content (Supplementary Fig. 2D). This also demonstrates a relative lack of platelet-single nucleated cell events that could be considered platelet-leukocyte aggregates (Supplementary Fig. 2B). Expression of one antigen of interest was examined at a time, with differences assessed between treatment groups and organ niches.

Expression of MHC class I on the surface of MKs was not affected by the sensitisation state of the animal alone, with no significant differences detected in the lung or BM-resident MKs between the two treatment groups. However, BM- resident MKs had a smaller proportion of cells expressing MHC class I compared to the HDM extract treatment group (Fig. 3D).

MHC class II expression on MKs was found to be dependent on both organ niche and sensitisation state. More of the lung-resident MKs expressed MHC class II in comparison to the BM-derived MKs in both PBS and HDM extract treatment groups. Moreover, the sensitisation state to HDM extract significantly increased the amount of MHC class II-positive MKs in the lung, but not in the BM. No effect of the repeated allergen treatment was detected on the frequency of MHC class II-positive MKs harvested from the BM (Fig. 3E).

The expression of FcεRIα was also influenced by both organ niche and sensitisation state. While no differences between lung and BM-derived MKs were detected in the PBS treatment group, a significant increase in the frequency of lung-resident FcεRIα -positive MKs versus BM-derived MKs was observed in the HDM treatment group. Furthermore, the sensitisation state to HDM extract resulted in a significant doubling in expression of FcεRIα on lung-resident MKs when compared to the control treatment group. The frequency of FcεRIα -positive MKs harvested from the BM was not affected by the sensitisation state to HDM extract (Fig. 3F). Finally, expression of CD40 was found to be low and comparable across MKs in both organ niches, and was not affected by the sensitization state (Fig. 3G). However, it should be noted that CD40 -positive events were identified based on the CD41-postivity alone.

**Fig. 4:**
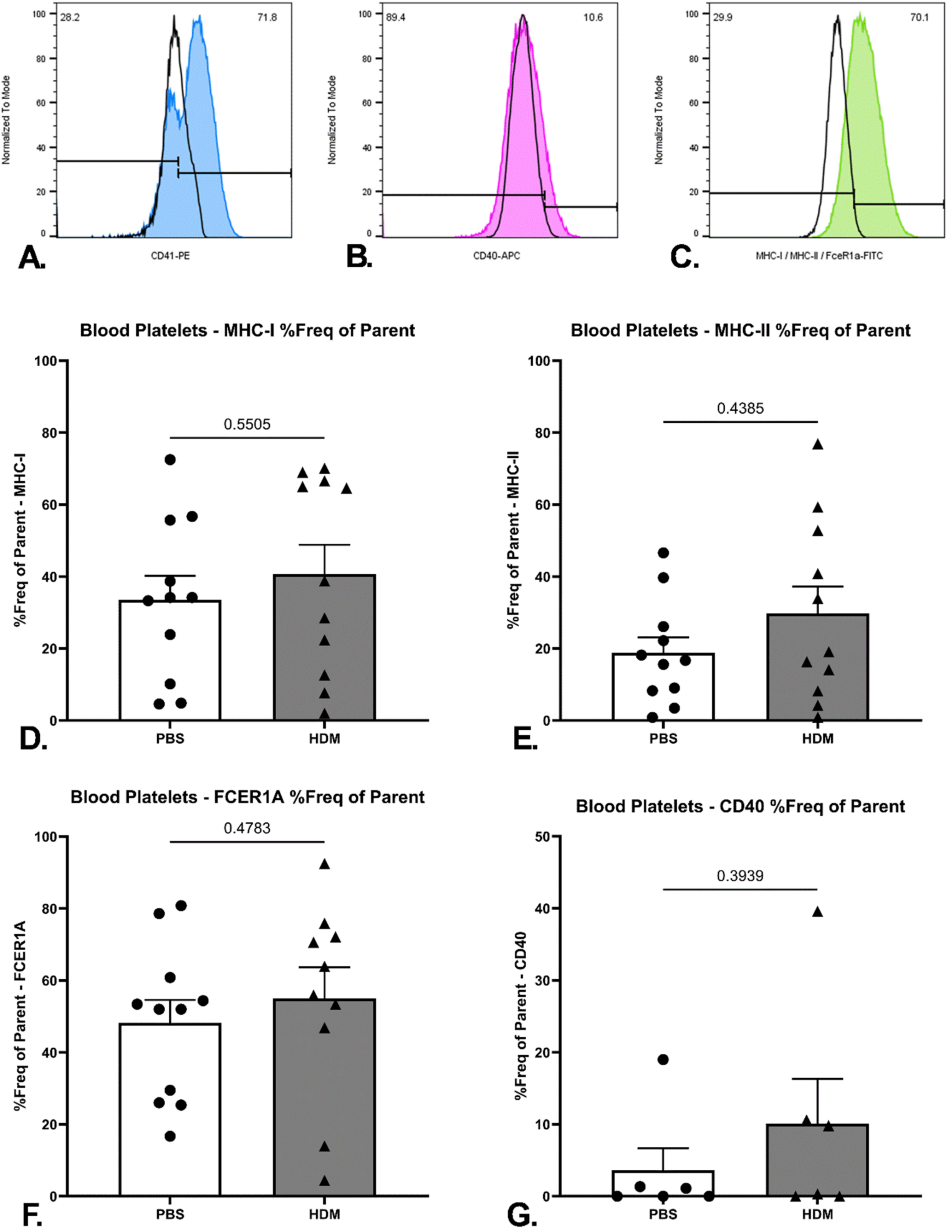
Sequential flow cytometry panels demonstrating the gating strategy applied to identify antigen-of-interest-positive mouse circulating blood platelets. Platelets were identified by size based on forward scatter-area (FSC-A) versus side scatter area (SSC-A) axes and subsequently confirmed for PE-CD41 positivity (blue histogram) against a triple isotype control staining condition of the same sample (black solid line) (**A**), before examination for APC-CD40 (green histogram) (**B**), FITC- MHC class I, MHC class II or FcƐRIα (**C**), against a double isotype control staining condition of the same sample (black solid line). Percentage of circulating platelets with surface expression of MHC class I (*n* = 11 per group) (**D**), MHC class II (*n* = 11 per group) (**E**), FcƐRIα (*n* = 10–11 per group) (**F**), and CD40 (*n* = 6 per group) (**G**) in ACD-anticoagulated blood samples of mice treated with PBS or HDM extract. Samples were collected via cardiac puncture on Day 14, 24 h the last PBS or HDM extract dose administration. Data expressed as mean +/− SEM (*p* < 0.05, Mann-Whitney test). Data is from three independent experiments, except for CD40 expression, which was obtained from one experiment. Each data point represents an individual mouse

To determine whether the sensitisation state of the animal affected the expression of the same markers of interest on the surface of circulating blood platelets, blood samples from the systemic circulation were used to assess the frequency of platelets positive for MHC class I, MHC class II, FcεRIα and CD40 expression (Fig. 4A–C).

Although expression of all markers of interest was detected on the surface of circulating blood platelets, no statistically significant differences in the percentage frequency of the total platelet population expressing either MHC class I, MHC class II, FcεRIα or CD40 were detected between the control and HDM extract-treated animal cohorts (Fig. 4D–G). This could potentially be due to the transient nature of the platelet subpopulations, and therefore the endpoint of the current experimental design did not allow their detection. Additionally, the specialised antigen presentation-and processing platelet subpopulation size might be modest, and determined by progeny arising from lung resident MKs, making it more challenging to detect in the anticoagulated blood preparations. Finally, it is feasible for the specialised platelet subpopulations being recruited to the site of inflammation, in this instance the lung, that this consequently makes them undetectable in circulating blood.

### Platelets Differentially Co-localise with Antigen-Presenting Cells in the Lungs of Allergic Mice

**Fig. 5:**
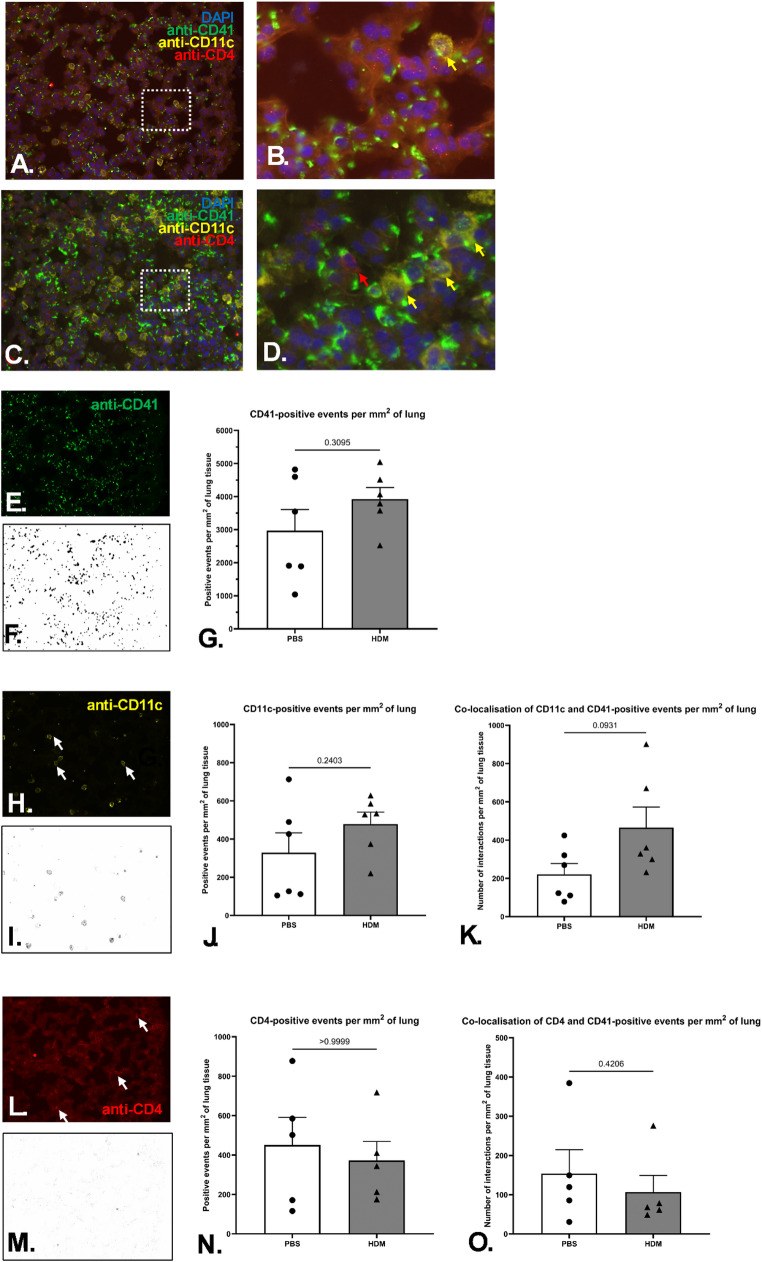
Co-localisation of platelets with immune cells in the lung. Immunofluorescent staining of snap-frozen murine lung was co-stained for anti-CD41 (Alexa 488; green), anti-CD11c (Alexa 647; yellow), anti-CD4 (Alexa 594; red) and DAPI (blue) taken from PBS treated (**A**,** B**), and HDM extract treated mice (**C**,** D**). The original images (**A**,** B**), taken at x20 objective magnification had sections identified (dotted boxes) that were expanded to twice the size and brightness raised by 40% using the Powerpoint ‘Format Picture Tool’ in order to visualise the cell interactions (**C**,** D**). The generated images were split into separate channels **(E**,** H**,** L)** and deconvoluted **(F**,** I**,** M)** to allow for positive events quantification per mm^2^ of lung tissue individually for CD41-positive (*n* = 6 per group) **(G)**, CD11c-positive (*n* = 6 per group) (**J**), and CD4-positive (*n* = 5 per group) events (**N**). Co-localisation of CD41-positive events with CD11c-positive events (**K**) and CD4-positive events (**O**). Images were analysed in ImageJ. Six fields of view at 20x magnification were selected in a random manner, and a mean value for each animal tissue was calculated per individual marker, and the respective co-localisation occurrence. Each datapoint corresponds to the individual animal mean for the respective readout. Data presented as group mean +/− SEM (*p* < 0.05, Mann-Whitney test). Data is from two independent experiments. Each data point represents an individual mouse

Next, we aimed to investigate whether CD41-positive platelet infiltration into the lung was increased on the establishment of the allergic pulmonary phenotype evoked by HDM extract, as well as whether platelets that have accumulated in the lung had co-localised with CD11c-positive antigen-presenting cells (APCs) and CD4-positive T cells. Identification of individual and co-localized cells with platelets is shown in Fig. 5A–D. Evidence of platelet co-localization with CD11c-positive cells, and to a lesser extent, CD4-positive cells can be observed lung tissue from HDM extract-exposed mice (Fig. 5C, D) compared to PBS treated controls (Fig. 5A, B). Whilst no difference in the frequency of single CD41-positive (Fig. 5G) and CD11c-positive (Fig. 5J) events was noted in the lung parenchyma after HDM sensitisation and exposure, the frequency of the platelets and CD11c-positive co-localisation events within the lung tissue doubled in the HDM extract-treated animals compared to PBS treated controls (Fig. 5K).

No statistically significant changes in the number of single CD4-positive events were observed in the lungs of PBS and HDM extract-treated animals (Fig. 5N). Furthermore, these CD4-positive events were not found to more frequently colocalise to CD41-positive platelets in the sensitised animals compared to non-sensitized control animals (Fig. 5O).

Taken together these observations suggest that the co-localisation of both CD41-positive platelets and CD11c-positive APCs in response to an allergic inflammatory stimulus increases, suggesting a biologically relevant interaction in terms of allergen sensitisation and antigen presentation. It can also be concluded that the increase in the colocalization frequency between platelets and APCs within the lung tissue on sensitisation of HDM extract is not purely due to the increased frequency of CD41-positive events in the lung, as no similar effect was observed in terms of colocalization with CD4-positive events.

**Fig. 6:**
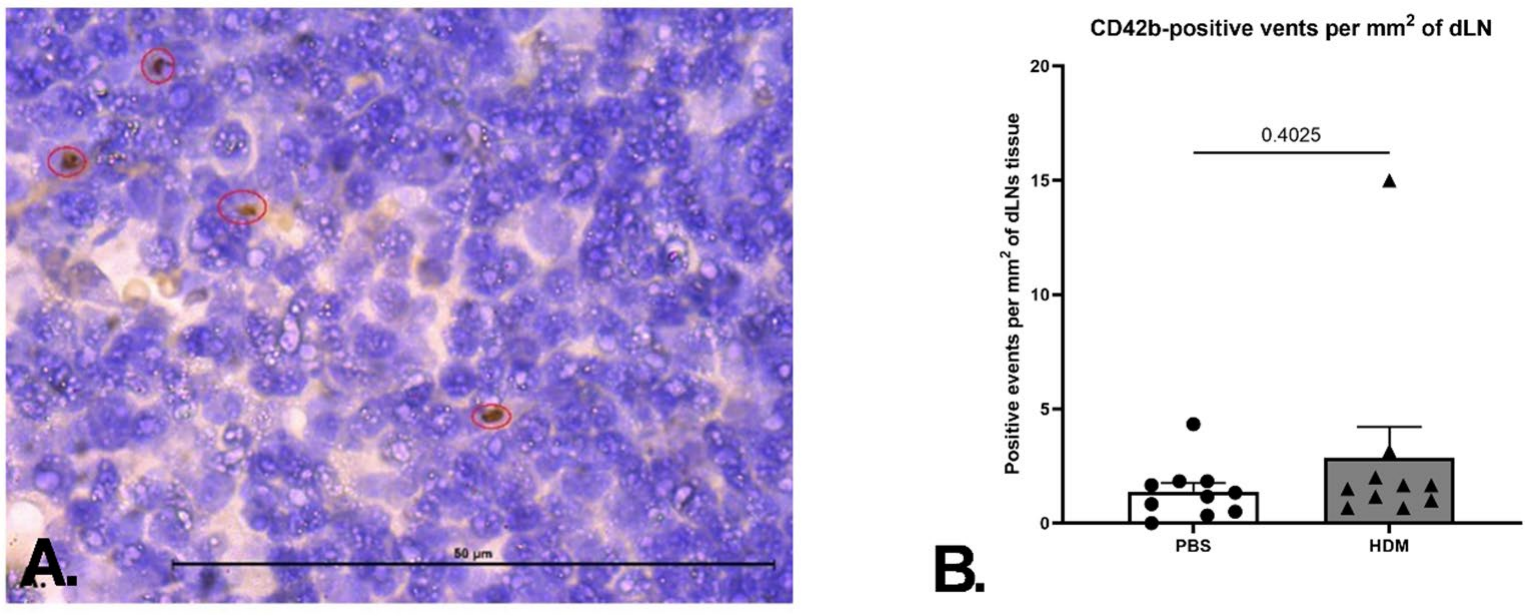
Platelets detected in the dLNs tissue utilising the DAB staining technique for the CD42b marker expression on day 14 (*n* = 10 per group), representative image (**A**). Tissue was excised 24 h after the last intranasal dose administration. Six fields of view at 100x magnification were selected in a random manner, and a mean value for each animal tissue was calculated (**B**). Each datapoint corresponds to the individual animal mean for the respective readout. Data presented as group mean (*p* < 0.05, Mann-Whitney test). Data is from two independent experiments. Each data point represents an individual mouse

To evaluate the possibility of platelets facilitating the migration of mature APCs into the lymph nodes for the downstream antigen processing and presentation, the dLNs of control and HDM extract -sensitised animals were excised and stained for CD42b for platelet detection. It was observed that platelets are not present in large numbers in PBS-treated mice, and no statistically significant changes were observed following HDM extract sensitisation (Fig. 6A, B). Given these two observations, it may be concluded that platelets either do not have an ability to migrate to dLNs, or that the process requires a temporal analysis after initial sensitization.

## Discussion

Although a substantial body of historical data describes the effects of temporal thrombocytopenia on the manifestation of a Th2-skewed response in vivo [1–8], most studies have limited translational relevance. This is due to several factors, including the use of allergens that are not airborne allergens in patients with allergic airway disease in humans (e.g. chicken egg white- Ovalbumin protein; OVA), clear temporal separation between the sensitisation and challenge phases, routes of administration poorly mimicking human exposure (e.g. intraperitoneal rather than airway), and the inclusion of immunogenic adjuvants such as aluminium hydroxide in the allergen preparations used for sensitisation.

This study is the first to demonstrate the requirement of platelets during the sensitisation phase to a clinically relevant allergen, administered via the respiratory tract to establish an allergic phenotype *in vivo.* The platelet depletion strategy employed in the current model of allergic pulmonary inflammation induced by HDM extract is unique in its timing and scope with regard to repeated respiratory exposure.

Directional transmigration of platelets occurs both in vitro and in vivo, including their migration to the lungs in response to HDM extract exposure being IgE- and CCR3-dependent [11, 14]. In this same model, we and others have previously reported the extravascular accumulation of platelets around the airways and parenchyma [14], after sensitization and exposure to ovalbumin [11], or Alternaria extract [31]. A consequence of the temporary platelet depletion during the sensitisation phase in the current model was a reduction in the abundance of pulmonary platelets defined as anucleate CD42b-positive events on day 5. Although we did not distinguish between the intra- and extravascular localisation of platelets, this observation indicates that platelet depletion was not constrained to the systemic circulation alone. It can therefore be suggested that platelet-mediated effects in terms of the development and establishment of the pulmonary allergic phenotype can occur either systemically and/or locally. It should also be noted that the systemic administration of platelet-depleting antibodies against GP1bα has been reported to partially deplete lung, but not BM MKs [17]. It is therefore possible that this also occurred in our studies, and that the impact of administering the GP1bα antibody on reduction of allergic inflammation might also be caused through local depletion of lung resident MKs. However, the situation is currently unclear because the pulmonary recruitment and migration of platelets from the circulation also occurs during localised allergen exposure to the lungs, which can itself induce a systemic response, as is evident from the intranasal sensitisation to HDM extract being able to elicit platelet recruitment to non-pulmonary vascular beds when discretely challenged with allergen application [11, 14].

The triad of markers - total plasma IgE, and BALF IL-4 and IL-13 – together with quantification of systemic and local eosinophil counts, was used to characterise the kinetics of the allergic phenotype development and the subsequent response to allergen exposure in the lung. The absence of a statistically significant increase in these parameters in platelet-intact HDM extract -treated animals compared with their respective controls at day 5 indicates that the period of experimentally induced thrombocytopenia was confined to the sensitisation phase. In contrast, the statistically significant elevation of all these markers at the final experimental time-point at day 14 confirmed successful sensitisation to HDM with a subsequent allergic pulmonary phenotype in mice with an intact platelet population. Furthermore, the pharmacokinetic profile of the anti-GP1bα (CD42b) platelet-depletion antibody allows us to conclude that the platelet population would have been restored by day 7, 48 h after the last dose of the antibody [7, 25, 26].

As IL-4 and IL-13 production leads to activities that tightly regulate IgE production by B cells [32–34], their initial production must originate from an early, IgE-independent source to initiate and prime the type I hypersensitivity response. The marked reduction in IL-4 and IL-13 levels observed in platelet-depleted mice indicated that platelets act at the very beginning of the process of allergen sensitization, before IgE-class switching and IgE-mediated response phase can occur. The absence of scientific evidence of the ability of either platelets or their progenitor MKs to produce these cytokines therefore leads to the suggestion that, in this instance, the MK/platelet axis must act as enablers or amplifiers of IL-4 and IL-13 production by other immune cells, and we have previously reported a reduction in IL-4 production in mice systemically sensitized to OVA and similarly made thrombocytopaenic during the period of sensitization [7].

 In vitro and in vivo findings demonstrate that CD4 + T cells are likely to be the sole source of IL-4 production required for IgE synthesis by B-cells, with levels produced being sufficient for Th2 differentiation [35, 36]. To examine whether the MK/platelet axis could influence the production of IL-4 through a direct cell-to-cell interaction with CD4 + cells in the lung, we have assessed whether the co-localisation frequency of pulmonary CD4 + events and CD41 + events increased in response to allergen sensitisation. The absence of significant increases in CD4 + events frequency alone or their co-localisation with CD41 + events in the lung after repeated allergen exposure suggests that the MK/platelet axis involvement in the sensitisation process is likely to take place before T cell expansion and production of Th2 cytokines; however this requires further investigation.

Although investigated in the present experiments, type 2 innate lymphoid cells (ILC2s) have been proposed as an alternative pulmonary source of IL-4 and IL-13 [37]. Predominantly localised within the mucosal layers, ILC2s exhibit Th2-like functionality, secreting canonical mediators including IL-4 and IL-13 upon activation by epithelial-derived alarmins such as IL-33. *Der P I*, a major allergenic protein within the HDM extract, possesses cysteine protease activity [38], and occludin within the airway epithelial tight junctions serves as one of its enzymatic substrates [39]. It is therefore reasonable to infer that epithelial damage induced by HDM proteases in the current model occurs, but may be transient, likely limited to the period immediately following intranasal administration of allergen, particularly during the initial exposures. The resulting release of alarmins, including IL-33, could stimulate pulmonary ILC2s to produce IL-4 and IL-13, thereby promoting B cell activation, Ig class switching, and ultimately the establishment of a Th2-skewed immune response [40]. It is therefore interesting to note that IL-33 can modulate the platelet proteome, function and biogenesis; and is mechanistically involved in platelet-driven eosinophilia in response to papain [41, 42].

The differentiation of naïve CD4-positive T cells into the Th2-type has been reported to be promoted by conventional dendritic cells subclass 2 (cDC2) [43]. Characterised by relatively lower migratory properties in comparison to cDC1s [44], it is feasible that the T cell differentiation at least partially occurs within the murine lung [45]. Here we observed an increased frequency of interaction between the single anucleate CD41-positive and CD11c-positive events within the lungs of HDM-sensitised animals, but not CD11c-positive events alone. Although it is not possible to distinguish between the pulmonary cDC subclasses, nor the general APC population, the increased frequency of co-localisation between platelets and APCs highlights the potential physiological importance of the interaction between DCs and platelets in vivo. It can be proposed that this interaction may potentiate DC maturation and / or differentiation within the pulmonary organ niche, therefore indirectly affecting the processes of antigen internalisation and presentation, with the subsequent effect on the activation of local CD4-positive T cells. Future experiments co-incubating allergen-exposed platelets or MKs with DCs may help understand the mechanism by which DCs become activated, proliferate, or undergo maturity by platelets, and determine if the MK/platelet axis has the ability to uptake and process antigen or release Th2 cytokines via blocking experiments with MHC class II inhibition or FcεRIα pathway interference to establish mechanistic dependence.

The present findings demonstrate that the expression of antigen-presentation proteins on MKs in the lung and BM is differentially regulated during the establishment of the HDM-induced allergic pulmonary phenotype. Specifically, lung-resident MKs exhibited a higher baseline frequency of MHC class I- and MHC class II -expressing cells compared to BM-derived MKs. Moreover, the increased frequency of MHC class II-positive MKs in the lungs of HDM-sensitised animals relative to the PBS-treated control groups suggests that the lung MKs may have the capacity to directly present HDM-derived peptides to CD4-positive T cells in a MHC class II -dependent manner [46]. Expression of FcεRI has been reported on a MK cell line and platelets allowing for the direct interaction of MKs with the allergen-specific antibodies [47]. In the current study, animals sensitised to HDM displayed a significantly higher proportion of FcεRIα-positive MKs in the lung, but not in BM, with levels of FcεRIα-positive MKs being consistent across the two examined organ niches of the PBS-treated cohort.

Collectively, these data indicate that the organ niche shapes the distinct pathophysiological roles of MKs, with lung-resident MKs exhibiting enhanced immunomodulatory functions linked to antigen presentation and allergen sensitization [17, 18], and direct interaction with IgE. This supports the concept that the immune phenotype of MKs is influenced by the organ microenvironment and local inflammatory milieu, with lung MKs adopting a more immunologically active profile, as would be expected in an organ continuously exposed to environmental antigens.

The circulating blood platelets, on the other hand, unexpectedly failed to demonstrate an increase in the cell frequency expressing MHC class I, MHC class II, FcεRIα, although showing consistent and sizeable single-positive proportions of the total population. The absence of the statistically significant increase may be explained by several factors including a modest size and transient nature of the subpopulation, their migration to the lung tissue, or high baseline receptor numbers. Additionally, it still remains unknown whether the transcriptomic and proteomic contents of MKs is passed to their ‘daughter’ platelets fully or partially, therefore meaning that platelets would not necessarily possess the biological functions for each of the MK-expressed proteins. Moreover, it could be proposed that the final result of the direct interaction between antigens with MHC class I, MHC class II, and FcεRIα, expressed on lung MKs triggers the production of ‘local’ platelets, possessing an immune signature corresponding to the microenvironmental triggers faced by their progenitor cell. These platelets could therefore be more tailored to portray their immunomodulatory properties within the lung, without the requirement to ever go into systemic circulation, therefore constituting a small and difficult to detect platelet subpopulation.

Finally, to examine whether platelets possessed the ability to egress from the lung tissue into the dLNs for the subsequent putative antigen-presentation to the CD4-positive T cells, platelet numbers were assessed in this lymphoid organ. However, we have not detected an increase in the platelet infiltration into the dLNs, with the levels being consistently low across the control and allergen sensitised animal cohorts. We recognise this data is limited to a single time point, and a time course analysis is required to fully understand the dynamics of possible platelet movement with antigen bearing APCs to the dLNs.

In conclusion, the currently described in vivo studies demonstrate the multifaced and bidirectional relationship between platelets and their progenitor MKs with the allergic pulmonary phenotype to HDM. The presence of the circulating blood platelet pool was shown to be an absolute requirement for the manifestation of a Th2-phenotype, indicative of a role for a MK/platelet axis in the early stages of sensitisation. On the other hand, on establishment of the allergic pulmonary phenotype, lung-resident MKs demonstrated immunophenotypical differences when compared to the BM-resident MKs, leading to a hypothesis that the MK/platelet axis may possess immunomodulatory functions at the commencement of antigen processing and presentation.

## Electronic Supplementary Material

Below is the link to the electronic supplementary material.

Supplementary Material 1

## Data Availability

No datasets were generated or analysed during the current study.

## Abbreviations

APCs: Antigen presenting cells
dLNs: Lung–draining lymph nodes
HDM: House dust mite
MK: Megakaryocyte
