# Supplementary material for: The Platelet/Megakaryocyte Axis is Necessary for Allergic Sensitisation and Inflammatory Responses to House Dust Mite in the Lung
Source: Lung. 2026 May 19;204(1):30. doi: 10.1007/s00408-026-00896-w (PMC13186874; doi:10.1007/s00408-026-00896-w)
Supplement: Supplementary file 1 — Supplementary Material 1 [file 408_2026_896_MOESM1_ESM.docx]

**Supplementary File**

**The platelet/megakaryocyte axis is necessary for allergic sensitisation and inflammatory responses to House Dust Mite in the lung.**

**Running Title:** Platelets and MKs in allergic airway inflammation

**Keywords:** Platelets, megakaryocytes, HDM, sensitisation, IgE, IL-4, IL-13, MHC Class II, antigen-presenting cells

Anna Chalidou, PhD^1^, Katie-Marie Case MSc ^1^, Carl Hobbs ^2^, Clive P Page, PhD ^1^, Simon C Pitchford, PhD ^1^.

^1^Pulmonary Pharmacology Unit, Institute of Pharmaceutical Science, Waterloo Campus, King’s College London, SE1 9NH

^2^Wolfson SPaRC (Sensory, Pain and Regeneration Centre), Institute of Psychiatry, Psychology & Neuroscience, Guy’s Campus, King’s College London, SE1 1UL

1. *Flow cytometry*

Blood samples were obtained via cardiac puncture into ACD tubes (Sigma, C2831) for flow cytometric analysis of platelets. A 50µl aliquot was stained with antibodies at a 1:10 dilution, where samples were stained for either a PE-anti-mouse CD41 antibody alone (BD, 558040) or in combination with one of the following: FITC-anti-mouse MHC class I (Invitrogen, 11-5998-82), FITC-anti-mouse MHC class II (Invitrogen, 11-5322-82), FITC-anti-mouse FcεRIα (TONBObiosciences, 35-5898-U100) or APC-anti-mouse CD40 (Invitrogen, 17-0401-82), and later lysed with an erythrocyte lysis solution.

Lungs were dissected out of the animals and cleared of the surrounding connective tissue. Right lungs were digested by mechanical and enzymatic (0.13mg/ml Liberase, Merck, 540-102-00-01; 20U/ml DNase I, Invitrogen, 18047-019) means. Both femurs were excised and the bones were washed out with 1ml of cold 1xPBS. The generated pellets were treated with an erythrocyte lysis buffer and washed to produce clear single-cell suspensions. 100µl aliquots of the generated samples were stained as described above with an addition of an APC-TOPRO-3 DNA stain (Invitrogen, T3605) to demonstrated ploidy of the CD41-positive events and confirm them as MKs.

1. *Histopathological examination of tissue*
   1. *Immunofluorescence*

Left lungs were excised, embedded in OCT (VWR Chemicals, 361603E), and snap-frozen in 2-methylbutane (Sigma-Aldrich, M32631) cooled with liquid nitrogen. Sections (7-10 µm) were cut using a Bright Model OT cryostat, mounted on Superfrost Plus slides (Epredia, J1810AMNZ), and air-dried overnight. Slides were fixed in 3.7% paraformaldehyde (PFA, Sigma-Aldrich, 8187081000) for 15 minutes, rinsed with ddH_2_O, and blocked with 1% bovine serum albumin (BSA, Sigma-Aldrich, A2153) in PBS.

Sections were incubated overnight at 4^O^C with anti-mouse CD41 (1:200, antibodies.com, A85853). Anti-CD4 (1:200, Abcam, ab183685), and Anti-CD11c (1:1000, Invitrogen, 14-0114-82) antibodies, followed by a 2 hour incubation at room temperature with Alexa Fluor 488 anti-rat (1:500, Invitrogen, A11006), Alexa Fluor 594 anti-rabbit (1:500, Life Technologies, A11037), Alexa Fluor 647 anti-hamster (1:500, Invitrogen, A21451), and DAPI. Slides were washed and mounted with Mowiol (Sigma-Aldrich, 81381).

Images (6 fields per lung, blinded) were acquired at 20x magnification using a Zeiss Axioplan 2 LED fluorescence microscope (Axiocam MRm, Axiovision v.4.8). Channels for DAPI, Alexa Fluor 488, 594 and 647 were merged. ImageJ (NIH) was used to quantify CD41-positive, CD11c-positive, and CD4-positive events (particles/mm^2^). Co-localisation of CD41 with CD11c or CD4 events was assessed by overlaying respective single-channel images and manually identifying interactions.

- 1. *DAB staining*

Tracheotomised lungs were inflated with 0.5mL 3.7% PFA, tied off, and fixed for at least 24 hours. Tissue was dehydrated, cleared, and paraffin-embedded using a Shandon Citadel 2000 processor. Sections (5 µm) were mounted on Superfrost Plus slides, dried overnight, and baked at 60^O^C for 1 hour. Slides were deparaffinised in xylene, rehydrated through graded IMS, and subjected to HIER in sodium citrate buffer (10 mM, 0.05% Tween 20, pH 6.0) for 11 minutes.

Endogenous peroxidase was quenched with 3% H_2_O_2_ in ethanol for 10 minutes. Sections were blocked with 1% BSA in PBS, then incubated for 2 hours with rabbit anti-mouse CD42b (1:200), followed by 1 hour with biotinylated goat anti-rabbit IgG (1:200) and avidin-biotin-HRP complex (Vector, PK1600). DAB substrate (1.2 mM in 0.1 M Tris, 0.3% H_2_O_2_, pH 7.6) was applied for 10 minutes, followed by haematoxylin counterstain (Sigma-Aldrich, H3136). Slides were dehydrated, cleared and mounted with Dibutylphthalate Polystyrene Xylene (DPX, Sigma, 6522).

Images (5-6 fields per mouse, blinded) were acquired at 20x using a Leica DM2000 LED microscope (DFC295 camera, LAS v4.4). DAB signal was quantified in ImageJ using the Colour Deconvolution plugin.^1^ Thresholds were adjusted to optimise signal-to-noise, and particles corresponding to platelet-sized objects (≈ 3.1 pixles, 0.5 µm radius) were quantified.

For lung-draining lymph nodes (dLNs), thoracic plucks were dissected, and tissues were processed as above for platelet detection.

1. *Broncho-alveolar lavage procedure and cell counting of BALF and blood*

Animals were culled by anaesthetic overdose (0.2ml of 25% urethane, intraperitoneally) on days 5 and 14, 24 hours after the last intranasal (i.n.) administration of HDM extract. The trachea was exposed and cannulated, and 3 aliquots (0.5 mL each) of warm, sterile saline solution were injected into the lung; the resultant fluid was drawn out with a 1-mL syringe and put on ice. Total cell counts were obtained by adding 50 μL of bronchoalveolar lavage fluid (BALF) to 50 μL of hemolysis solution (0.1% methylene blue in 1% acetic acid). Samples were counted on an improved Neubauer haemocytometer with a 20× objective using a Zeiss upright microscope.

Differential cell counts were obtained from cytospin preparations of BALF stained with the DiffQuick system (Gamidor Ltd) to differentiate mononuclear cells, neutrophils and eosinophils and 200 cells were counted per sample using a 20 × objective using a Zeiss upright microscope.

Blood (5 μL) was collected before challenge by tail bleed, using a pipette. The blood was added to 95 μL Stromatol solution (Mascia Brunelli Srl, Milan, Italy) for platelet counting using an improved Neubauer haemocytometer) under a 40 × objective using a Zeiss upright microscope. Total and differential blood cell counts were performed as previously described.^2,3^

In all instances of cell counting (BALF, blood), the experimenter was blinded to sample identity.

1. *Enzyme-Linked Immunosorbent Assays (ELISAs) for systemic and local biomarkers*

Systemic blood was collected via cardiac puncture, mixed with 150 µL acid-citrate-dextrose (ACD; Sigma, C3821), and centrifuged to obtain plasma. Plasma was diluted at a 1:25 ratio and analysed for total IgE using an ELISA (Invitrogen, 88-50460), following the manufacturer's instructions.

Neat BALF was used to quantify IL-4 (Invitogen, 88-7044), IL-13 (Invitrogen, 88-7137) and IL-33 (Invitrogen, 88-7333) concentrations by marker-specific ELISAs, as per manufacturer’s protocols.

Plates were read at 450 and 570 nm using a SpectraMax 384 Plus plate reader (Molecular Devices). Absorbance at 570 nm was subtracted from 450 nm for signal correction, and background values (blank well signal) were removed. Protein concentrations were determined from standard curves.

1. Landini G, Martinelli G, Piccinini F. Colour deconvolution: stain unmixing in histological imaging. Bioinformatics. 2020; 37: 1485-1487.
2. Pitchford SC, Yano H, Lever R, Riffo-Vasquez Y, Ciferri S, Rose MJ, Giannini S, Momi S, Spina D, O'connor B, Gresele P, Page CP. Platelets are essential for leukocyte recruitment in allergic inflammation. *J Allerg Clin Immunol.* 2003; 112: 109–118.
3. Pitchford SC, Momi S, Giannini S, Casali L, Spina D, Page CP, Gresele P. Platelet P-selectin is required for pulmonary eosinophil and lymphocyte recruitment in a murine model of allergic inflammation. *Blood*. 2005 105: 2074–2081.

**Supplementary Figure 1**





**Supplementary Figure 1.** Time course of circulating leukocyte numbers following platelet depletion and allergen sensitisation. **(A-C)** Circulating total cell counts of Days 1, 5 and 14 (n= 12, 6, and 6 per group, respectively). **(D-F)** Circulating neutrophil counts of Days 1, 5 and 14 (n= 12, 6, and 6 per group, respectively). **(G-I)** Circulating mononuclear cell counts of Days 1, 5 and 14 (n= 12, 6, and 6 per group, respectively). Samples on Day 1 and 5 were collected 24 hours after the last R300 dose administration (i.v.). Samples on Day 14 were collected 24 hours after the last HDM dose administration (i.n.). Data presented as mean +/- SEM (p< 0.05, 2-Way ANOVA with Uncorrected Fisher’s LSD).

**Supplementary Figure 2**





**Supplementary Figure 2.** Analysis of ploidy content of cells harvested from lungs. **(A)** Identification of CD41-positve events in the single cell suspension. TOPRO positivity to reveal cells with 2n and 4n ploidy shown in the: (**B**) CD41-negative events gate and (**C**) CD4-positive events gate. (D) Quantification of TOPRO mean fluorescent intensity (MFI) in the ≥2n region comparing cells with and without CD41 positivity. Data presented as mean +/- SEM (p< 0.05, T-test).
